# Evaluating the scaling up of an effective implementation intervention (PACE) to increase the delivery of a mandatory physical activity policy in primary schools

**DOI:** 10.1186/s12966-023-01498-y

**Published:** 2023-09-06

**Authors:** Alix Hall, Cassandra Lane, Luke Wolfenden, John Wiggers, Rachel Sutherland, Nicole McCarthy, Rebecca Jackson, Adam Shoesmith, Christophe Lecathelinais, Penny Reeves, Adrian Bauman, Karen Gillham, James Boyer, Patti-Jean Naylor, Nicola Kerr, Nicole Kajons, Nicole Nathan

**Affiliations:** 1 School of Medicine and Public Health, The University of Newcastle, Locked Bag 10 Wallsend, NSW 2287 Newcastle, Australia; 2 Hunter New England Population Health, Hunter New England Area Health Service, Newcastle, NSW Australia; 3 National Centre of Implementation Science, The University of Newcastle, Newcastle, NSW Australia; 4 Hunter Medical Research Institute, New Lambton Heights, NSW Australia; 5 School of Public Health, University of Sydney, Sydney, NSW Australia; 6 School Sport Unit, NSW Department of Education, Turrella, NSW Australia; 7 School of Exercise Science, Physical and Health Education, University of Victoria, Victoria, BC Canada; 8 Health Promotion, Mid North Coast Local Health District, Coffs Harbour, Australia; 9 Health Promotion and Population Health Improvement, Central Coast Local Health District, Gosford, NSW Australia

**Keywords:** School, Physical activity, Scale-up

## Abstract

**Background:**

*Physically Active Children in Education (PACE)* is an effective implementation intervention for increasing the number of minutes classroom teachers schedule physical activity each week. To date, evaluations of PACE have included a smaller number of schools from only one region in New South Wales Australia. If PACE is to have population-wide benefits we must be able to deliver this support to a larger number of schools across multiple regions. This study aimed to evaluate the scale-up of PACE.

**Methods:**

An uncontrolled before and after study, with 100 schools from three regions was conducted. Participating schools received PACE for approximately 12 months. We assessed the following outcomes: delivery of the evidence-based intervention (EBI) (i.e. minutes of physical activity scheduled by classroom teachers per week); delivery of the implementation strategies (i.e. reach, dose delivered, adherence and indicators of sustainability); and key determinants of implementation (i.e. acceptability of strategies and cost). Data were collected via project officer records, and principal and teacher surveys. Linear mixed models were used to assess EBI delivery by evaluating the difference in the mean minutes teachers scheduled physical activity per week from baseline to follow-up. Descriptive data were used to assess delivery of the implementation strategies and their perceived acceptability (i.e. PACE). A prospective, trial-based economic evaluation was used to assess cost.

**Results:**

Delivery of the EBI was successful: teachers increas their average minutes of total physical activity scheduled across the school week by 26.8 min (95% CI: 21.2, 32.4, *p* < 0.001) after receiving PACE. Indicators for delivery of implementation strategies were high: 90% of consenting schools received all strategies and components (reach); 100% of strategies were delivered by the provider (dose); >50% of schools adhered to the majority of strategies (11 of the 14 components); and acceptability was > 50% agreement for all strategies. The incremental cost per additional minute of physical activity scheduled per week was $27 per school (Uncertainty Interval $24, $31).

**Conclusions:**

PACE can be successfully delivered across multiple regions and to a large number of schools. Given the ongoing and scalable benefits of PACE, it is important that we continue to extend and improve this program while considering ways to reduce the associated cost.

**Supplementary Information:**

The online version contains supplementary material available at 10.1186/s12966-023-01498-y.

## Background

The World Health Organization (WHO) recommends that children aged between 5 and 17 years participate in an average of 60 min of moderate-to-vigorous physical activity (MVPA) per day [1]. International evidence suggests that for many countries less than 65% of children meet such guidelines [2, 3]. As schools provide continuous access to the majority of children, the WHO has recommended the implementation of school based policies which support children’s physical activity [4]. Accordingly, many countries have instigated policies that require schools to deliver a minimum amount of physical activity to students [5–9]. For example, the United Kingdom and parts of Canada and the United States require schools to schedule physical activity amounting to between 120 and 150 min per week. In New South Wales (NSW) Australia, the Department of Education currently requires schools to schedule a minimum of 150 min of planned moderate, with some vigorous, physical activity across the school week [10]. Planned physical activity can consist of any of the following components: physical education (PE), sport, or other structured activities such as integrated lessons or short bouts of classroom activity known as “energisers”.

Despite such policies, international research suggests that most schools fail to routinely implement physical activity policies [5, 7, 8, 11–14]. For instance, cross-sectional surveys of school teachers from several countries have indicated poor implementation of physical activity policies by schools. A 2013 survey of 136 teachers in Ontario, Canada, showed that 46% were not implementing the provincial policy for school day physical activity [11]. Similarly, a 2012 survey of 1,243 teachers in Utah, United States, found that 56% did not know about the respective state mandate for school day physical activity [12]. A 2021 survey of 76 teachers in Denmark found that a higher 90.5% were not delivering curricular physical activity as per the national mandate [13]. In NSW Australia, 2021 data from 400 teachers showed approximately 70% of teachers were not scheduling the minutes of physical activity mandated in the state policy [14]. To ensure such policies have beneficial public health impact we need to identify ways to effectively support schools to implement physical activity policies.

However, there has been limited evidence as to the most effective strategies to support schools’ implementation of physical activity policies [15]. Subsequently, our research team have undertaken a series of randomised controlled trials to enhance the implementation of the NSW school physical activity policy (*Physically Active Children in Education* (PACE)) [14, 16, 17].

PACE, is a multi-strategy implementation intervention that supports teachers to increase their scheduling of classroom physical activity [14]. It consists of eight strategies: centralised technical assistance and ongoing consultation from an external support officer; principal’s mandated change to confirm commitment to the policy; identifying and preparing in-school champions who are school staff trained to support other staff from their school to schedule physical activity and support the delivery of PACE strategies; development of a formal implementation blueprint; educational outreach visits for school staff; distribution of educational materials; shared local knowledge (case studies of successful schools) on an online portal; and provision of a physical activity equipment pack [16]. In 2017, PACE was pilot-tested in a cluster randomised controlled trial (RCT) in 12 NSW Catholic primary schools (of 423 Catholic primary schools in the state) over a nine-month period [17]. Teachers at the six intervention schools received PACE. At nine-month follow-up teachers from intervention schools scheduled on average significantly more minutes of physical activity across the week compared to controls (36.6 min; 95% confidence interval [CI] 2.7, 70.5; *p* = 0.04), with the greatest area of improvement seen in the minutes that teachers scheduled short, classroom activity breaks (i.e., energisers [23.4 min; 95% CI 3.2, 43.6; *p* = 0.03]) [17]. On the basis of these promising findings, a fully powered clustered randomised implementation trial with 61 primary schools (~ 3% of NSW primary schools) was undertaken across one school year [16]. Teachers at intervention schools significantly increased their implementation of physical activity by an average of 44 min more than teachers from control schools (44.2 min; 95% CI, 32.8, 55.7; *p* < 0.001) [14]. Again, the greatest area of improvement was seen in teacher’s scheduling of energisers, with an intervention effect of 23.1 min (95% CI 16.5, 29.6; *p* < 0.001). Furthermore, promising results were observed with regards to the cost-effectiveness of PACE, with an incremental cost-effectiveness ratio of Australian dollar (AUD) $29 (95% uncertainty interval [UI] $17, $64) for every additional minute of weekly physical activity implemented per school [18]. This was considered acceptable from the health service provider perspective to achieve school implementation of the policy [18].

While these studies provide evidence of the feasibility, effectiveness, cost-effectiveness and acceptability of PACE, they have been conducted in a small sample of schools (relative to the population of schools), and included schools from only one local region – the Hunter New England region of NSW Australia. This region covers a large geographical area (approximately 130,000 km^2^), and includes a socioeconomically and geographically diverse population with a large number of primary schools (> 400) [14, 19]. If interventions are to achieve their intended population health benefits, they must be scaled-up across multiple regions that potentially consist of different characteristics and challenges, and remain effective in the process [20]. However, scale-up is a complex process [21], and translating an effective intervention from small, well-controlled research studies into real world contexts presents unique challenges [22]. Currently, few studies have evaluated the scale-up of school-based physical activity programs, with most studies focusing on efficacy evaluations conducted on a small number of schools under well-controlled conditions [23, 24]. To ensure the ongoing benefit of effective implementation interventions such as PACE, evaluations are required to determine whether they can be successfully delivered on a broader scale across multiple regions, under real-world conditions. Such evaluations will usually require the use of more pragmatic research designs, such as uncontrolled before-and-after studies, as the conduct of randomised controlled trials with large sample sizes and under real-world conditions become less feasible. Thus, the next step in the research-dissemination pathway for PACE is to assess whether it can be delivered on a larger scale (i.e., during scale-up) [25], and explore any issues that may impact its ability to be scaled such as its cost. Such an evaluation will provide important insight into the challenges and considerations for scaling-up effective implementation interventions designed to support the delivery of school-based physical activity.

Therefore, the current study aimed to evaluate whether the effective implementation intervention (PACE) could be delivered and evaluated to a large number of schools (100 schools vs. previous sample of 31 schools that received PACE in  effectiveness trial) across various diverse regions. We structured our analysis following the evaluation roadmap proposed by McKay et al. [25] for evaluating implementation during scale-up. Accordingly, we assessed both the (i) delivery of the evidence-based intervention (EBI) at scale (i.e. minutes of physical activity scheduled by classroom teachers); and (ii) delivery of the implementation strategies (i.e. PACE), including the indicators of: reach, dose delivered, adherence and sustainability. As a secondary aim we also assessed two key implementation determinants that impact scale-up: acceptability and cost of the implementation strategies. Given the significant implications cost has on whether an intervention can be successfully delivered at scale we also undertook an economic evaluation.

## Methods

### Ethics

Approval to conduct the study was obtained from the Hunter New England Human Research Ethics Committee (2019/ETH12353), The University of Newcastle Human Research Ethics Committee (Approval Number H-2008-0343) as well as the NSW Department of Education (SERAP no. 2,017,184) and the relevant Catholic Schools Offices.

### Design and setting

We conducted an uncontrolled before and after study of 100 schools across three different regions (i.e. Local Health Districts) in NSW: Hunter New England, Mid North Coast and Central Coast. Together these regions cover a large geographical area (more than 141,000 km^2^) and consist of a socioeconomically and demographically diverse population of approximately 192,500 children aged 5–14 years [26]. Each region has its own work force, infrastructure and budget dedicated to supporting schools delivery of chronic disease prevention programs, including physical activity.

### Participants and recruitment

#### Schools

All primary schools (those that cater for children aged 5–12 years), including government, Catholic, and independent (private) schools were eligible to participate, excluding those that were already involved in a physical activity trial, had already received PACE, or exclusively catered for children requiring special education needs. Eligible schools were identified from publically available lists of schools. The principal from eligible schools were sent an invitation email including an information sheet and consent form.

#### Teachers

All classroom teachers from consenting schools were invited to complete a pen-and-paper survey at baseline and at 12-month follow-up. Informed consent was assumed based on teacher completion of the study survey.

### Multi-strategy implementation intervention

As the aim of this study was to assess whether the effective PACE implementation intervention could be delivered on a larger scale across multiple regions, and explore any issues that may impact its scale-up, minimal adaptations were made to PACE. The strategies were developed to address identified barriers to teacher’s scheduling of physical activity [27]. They were: theoretically informed by the Theoretical Domain Framework [28] and Behaviour Change Wheel [29], based on extensive formative research, and co-designed in consultation with an advisory group that included experts in physical activity, education, implementation science and policy [16]. An overview of the final eight PACE implementation strategies are shown in Table 1.

**Table 1:** The implementation strategies of PACE and their dose delivered and adhered to by schools

Implementation strategy	Strategy content	Dose delivered by service delivery staff n (%)	Adherence (by schools) of implementation strategies n (%)
Centralize technical assistance and Provide ongoing consultation	Project officers (qualified as both a PE teacher and health promotion practitioner) provided technical assistance and ongoing consultation, including: telephone, email or in-person, to schools for the duration of the intervention period. This included supporting in-school champions to overcome barriers (by means of providing expert opinion as well as school-specific brain stormed solutions); and reviewing progress of the schools implementation plan and – if necessary – modification and re-setting of goals.	100 (100%)	88 (88%)
Mandate change	The following components were undertaken by project officers to help schools mandate change and support their implementation of the physical activity policy:		
	• A meeting with school principals and executives to highlight the importance of the policy	100 (100%)	99 (99%)
	• A request to school principals and executives to illustrate their support of the policy and communicate their expectations to the wider school community	N/A	58 (58%)
	• Support to schools to develop or amend their physical activity policy	100 (100%)	100 (100%)^a^
Identify and prepare champions	Each school nominated up to three in-school champions (existing teachers at the school) who drove the implementation of classroom physical activity in their school. In-school champions were the primary source of contact with project officers, who supported them to overcome school indifference and/or resistance that PACE may have provoked. In-school champions were encouraged to serve as role models to other school staff by engaging in the desired behaviours themselves.	100 (100%)	100 (100%)
	A one-day in-person training session, consisting of instructional and practical components, was delivered by project officers to in-school champions. Instructional learning included (a) education about the policy and the importance of physical activity for children, and (b) time to develop action plans requiring the identification of barriers/ facilitators to implementation and possible solutions to overcome these via an “if-then-what” plan. Practical learning included instruction and active participation in energisers, integrated lessons and examples of sport/PE lessons. Training was accredited by the state educational authority and provided contributed to teacher’s continuing professional development hours.	100 (100%)	100 (100%)
Develop a formal implementation blueprint	In-school champions were supported to develop a plan for the implementation of the policy in their school. The plan identified what the school aimed to specifically achieve, the strategies to do so, the resources available or required to implement the plan, and a timeline. Plans were broken into four school terms (over the course of one school year) to break up some of the more complex policy requirements into achievable tasks for in-school champions.	100 (100%)	100 (100%)^a^
Conduct educational outreach visits	Project officers or trained in-school champions conducted an in-person session with all teachers during a regular school staff meeting. During this session they: • Introduced the in-school champion(s) and communicated their role as the main point of contact and support for their school implementing the policy; • Provided information and education about the policy with a deliberate aim to reframe teachers’ perception of the policy from one that “adds to teacher load” to one that is “easily integrated into existing routines”. • Provided verbal persuasion about teachers capability to implement the policy; • Instructed and demonstrated examples of physical activity that teachers could incorporate into their classroom plan, such as energisers and PE lessons; • Prompted habit formation for some of the physical activity practices.	100 (100%)	78 (78%)
Develop and distribute educational materials	An “intervention manual” was provided to In-school champions. The manual included recourses they could use including, policy and timetable templates, exemplar physical activity timetables and PE curriculum schedules.	100 (100%)	100 (100%)
	Educational materials were provided to classroom teachers in the form of print and via the online portal, Such materials included practical games and strategies for increasing physical activity in lessons.	100 (100%)	94 (94%)
	The online portal also included professional learning videos that could be accessed by all teachers (including in-school champions). These videos reinforced the information received via the in-person educational outreach training.	100 (100%)	47 (47%)
Capture and share local knowledge	In-school champions were provided access to case studies from other schools. These case studies described examples of success stories from schools, explaining how in-school champions and teachers had overcome frequently reported barriers to implement the policy in their school. These case studies were part of the professional learning materials made available via the online portal.	100 (100%)	47 (47%)
Change physical structure and equipment	A general physical activity equipment pack was provided to each school by project officers – distributed to in-school champions at the one-day in-school champion training workshop. Equipment packs contained items to facilitate classroom physical activity, with examples including: balls, bean bags, “activity” cards. Examples of how these items could be used by classroom teachers were demonstrated at the in-school champion training day and via videos on the online portal.	100 (100%)	100 (100%)
	In-school champions were asked to support the development of classroom physical activity packs for all classrooms.	100 (100%)	38 (38%)

^a^This component was delivered during the in-school champion workshop and thus attendance at the workshop assumed adherence to this strategy

### Data collection

Baseline data collection occurred between December 2018 and April 2019. Follow-up data collection was conducted approximately 12-months later between October and December 2019. Consistent with the evaluation roadmap for the implementation and scale-up of physical activity interventions [25] we assessed outcomes at the following levels: EBI delivery (dose delivered), delivery of the implementation strategies and key implementation determinants. A description of the outcome measures and the relevant sources used to collect outcome data are described below and in detail in Table 2.

**Table 2:** Description of the outcomes measured according to McKay’s [25] implementation road map and the data sources each measure was obtained from

Outcome	Definition (25)	Data source: Method of assessment
Delivery of the intervention		
Dose delivered	The intended units of each component of physical activity that was delivered by classroom teachers	**Teacher surveys**: Teachers completed a daily logbook for one school week, whereby they recorded the minutes of physical activity they scheduled for each possible component of physical activity, including: sport, PE, energisers and integrated lessons.
**Delivery of implementation strategies**		
Reach	Proportion of intended schools who participated in the intervention	**Project records**: We aimed to recruit 100 schools to participate in PACE. The number of schools that took part and withdraw from the program were recorded by project officers. The number of schools that were exposed to each of the implementation strategies was also recorded.
Dose delivered	The intended units of each PACE strategy delivered by the PACE delivery team	**Project records**: Project officers recorded every project activity that took place for each individual school, including: • Records of emails/phone-call/ in-person visits between project officers and school stakeholders, including completed checklists of required content • Records of in-school champion workshops and teacher training, including completed fidelity checklists
Adherence	The extent to which schools implemented each PACE strategy as prescribed (i.e., with fidelity)	**Project records**: For each school, project officers recorded whether schools implemented each strategy as per protocol. For example: • If the school received the equipment pack, manuals, and other resources; • If the principal provided verbal commitment to undertake program and responsibilities expected of them; • If the in-school champion engaged in the communication with project officers (at least two occurrences of communication) to carry out tasks expected of them; • If teachers accessed the online portal and viewed professional learning videos (via portal analytics); and • If the in-school champion(s) attended a workshop and actively participated in activities
Sustainability (Maintenance)	Whether behaviour change by teachers is maintained	**Principal surveys**: Principals indicated (yes/no) whether they would continue to: • support teachers at their school to schedule physical activity required by the policy, • utilise the role of the in-school champion, and • provide PACE resources to teachers. **Teacher (in-school champion) surveys**: Teachers indicated (yes/no): • their willingness to continue with the PACE program and scheduling classroom physical activity, and • whether they had a succession plan for supporting delivery of physical activity following changes in key roles (i.e., handover system if they were to leave the school).
**Implementation determinants**		
Acceptability	Perceptions among teachers that PACE strategies were agreeable, palatable or satisfactory	**Teacher surveys**: Teachers indicated their perceived level of acceptability of PACE strategies via six questions (detailed in Table 4) using a five-point Likert scale (1 = strongly disagree, 2 = disagree, 3 = neither agree nor disagree, 4 = agree, 5 = strongly agree)
Cost	Money spent on the delivery of PACE from the perspective of the health service delivery provider	**Project records**: Project officers retrospectively recorded the costs associated with the delivery of implementation strategies. These records were coded by strategy and entered into an economic spreadsheet (as per protocol for PACE economic evaluations (18)).

#### Delivery of the evidence-based intervention (dose delivered)

The EBI of interest, which PACE was designed to support the delivery of, was the NSW Department of Education 150 min physical activity policy. In line with previous implementation trials evaluating PACE, EBI delivery (or dose delivered) was defined as teachers’ total minutes of scheduled physical activity across the school week. This was measured via a daily activity log-book included as part of a pen-and-paper survey completed by teachers at baseline and 12-month follow-up. This is the same method that was used in the pilot [17], effectiveness [14] and optimization trials of PACE [30] and as such the same data collection method was employed for this study. In the log-book teachers recorded the number of minutes they scheduled structured physical activity each day across one school week, including time in physical education (PE), sport or in-class physical activity such as integrated lessons or energisers. In previous trials this method of collecting teacher’s scheduling data had had high response rates (> 80%) [17]. Consistent with our previous trials [14, 16, 17], schedule data was considered eligible if: teachers provided five days of data (to ensure consistency with the weekly policy requirements we were assessing), and the total number of minutes did not exceed 250 min (to align with the time requirements for other key learning areas set out by the Department of Education) [31].

#### Delivery of implementation strategies (i.e. PACE)

At follow-up, we assessed the following outcomes relating to the delivery of the implementation strategies (i.e. PACE): reach, dose delivered, adherence, and indicators of sustainability (maintenance) (see Table 2 for a detailed description).

#### Implementation determinants

At follow-up we also assessed two implementation determinants that were considered important for understanding potential barriers that may impact the scale-up of PACE: acceptability and cost (see Table 2 for a detailed description).

### Sample size

A target sample of 100 schools was planned to provide 80% power to detect a difference of 9.2 min between baseline and follow-up in the mean minutes of weekly total classroom physical activity scheduled by teachers. The following were assumed in making this calculation: a baseline standard deviation of 45 min, an intraclass correlation coefficient (ICC) of 0.2 and an average of 13 teacher surveys returned per school [16].

### Analysis

#### Delivery of the evidence-based intervention (dose delivered)

Separate linear mixed models were used to assess the overall scheduling (i.e. minutes of total physical activity scheduled) and the individual components of scheduled physical activity (i.e. minutes of scheduled PE, sport, integrated lessons and energisers). Each model compared the difference in the mean minutes scheduled at baseline to follow-up, and included a random intercept for school, a random intercept for teacher to account for possible repeated measurements, and a random slope for time. Missing data were handled within the linear mixed models, which uses all available data assuming data are missing at random. Possible confounders were adjusted for by including them as fixed effects in the linear mixed regression models. School level confounders included: school: type (i.e. government, Catholic, independent), geographical location (i.e. rural or urban) and socioeconomic disadvantage classification. While teacher level confounders included: sex, employment status, years of teaching experience and whether they job share (i.e., a teacher who shares the teaching tasks of one class with another teacher). Two sensitivity analyses were undertaken: [1] including only schools who had valid data at both baseline and follow-up; and [2] adjusting for region in addition to the other adjustments. The unadjusted and adjusted mean difference, 95% CIs, and *p*-value from the adjusted models are reported.

#### Delivery of implementation strategies and implementation determinants

Descriptive statistics were used to analyse outcomes relating to delivery of the implementation strategies and for the determinant acceptability.

#### Cost analysis

A prospective, trial-based economic evaluation of PACE was conducted. A health care sector and modified societal perspectives were taken, with the societal perspective restricted to health care providers and schools as they represent those financially impacted by PACE. A time horizon of one-year was taken as this was consistent with the length of the trial. Costs are reported in AUD$2019/20. Costs associated with the delivery and resource use of the implementation strategies (i.e. labour and materials) were prospectively measured and recorded. The incremental cost of PACE was calculated as the cost to implement the PACE strategies, as it was assumed all costs were wholly incremental to usual practice. The total costs overall and those incurred by the health service and school separately were calculated, along with the average cost of delivering PACE per school. An incremental cost effectiveness ratio was calculated using paired cost and outcome data, and by dividing the incremental cost by the estimated effect of PACE on the total minutes of physical activity scheduled by classroom teachers. Nonparametric bootstrapping analysis with 1000 iterations was used to account for uncertainty, which were graphed on a cost-effectiveness plane.

## Results

### Delivery of the evidence-based intervention (dose delivered)

Eighty-eight schools contributed valid data to this outcome for at least one of the time-points and were included in the analysis (valid data from 84 schools at baseline and 73 schools at follow-up). Teachers significantly increased their scheduling of total physical activity by an average of 26.8 min per week (95% CIs: 21.2, 32.4; *p* < 0.001; *n* = 88 schools with valid data) following receipt of PACE. A significant increase was also observed in the minutes teacher’s scheduled for integrated lessons (4.6 min per week; 95% CI: 1.4, 7.8; *p* = 0.006; *n* = 85 schools with valid data) and energisers (20.6 min per week; 95% CI: 16.5, 24.6; *p* < 0.001; *n* = 88 schools with valid data). No statistically significant differences were observed for PE or sport (see Table 3). A sensitivity analysis was conducted analysing only schools who returned both valid baseline and valid follow-up data (*n* = 69); the results were consistent with the primary analysis with no changes to the conclusions (see Supplementary Table 1). A second sensitivity analysis was conducted controlling for region; again the results were consistent with the primary analysis (see Supplementary Table 2).

**Table 3:** Characteristics of teachers who completed either the baseline or follow-up survey

Characteristic	Survey sample *n* = 790
	Frequency^a^ (%)
**Age (mean (SD))**	40 (10.95)
**Years teaching (mean (SD))**	13.78 (10.27)
**Sex**	
Male	123 (16%)
Female	623 (84%)
**Employment status**	
Permanent full-time	623 (85%)
Temporary full-time	106 (15%)
**Grade level taught**	
Infants only	260 (37%)
Primary only	380 (54%)
Infants and primary	39 (5.5%)
Other only (e.g. special education)	27 (3.8%)
**PDHPE specialist teacher**	16 (2.2%)
**Share teaching role**	219 (30%)
**In-school champion**	94 (12%)

^a^Cell totals may not equal total sample size due to missing data

### Delivery of implementation strategies (i.e. PACE)

#### Reach

From the 391 eligible schools, our target sample was 100 schools. We accepted 111 schools to receive PACE. However only 100 schools were included in the evaluation as 11 schools did not consent or have approval to be part of the evaluation and data collection component of the study. Most participating schools were from the Hunter New England region (74%), followed by Mid North Coast (18%) and Central Coast (8%), which roughly reflects the distribution of all schools across these three regions (71%, 16% and 13%, respectively). From the 100 participating schools, 89 had at least one teacher return a survey (partially or fully completed) at any of the two time-points, with 87 schools contributing at least some data relating to the outcomes and variables included in this study at baseline data collection and 80 schools at follow-up.

From these 100 schools, a total of 790 teachers were exposed to PACE and returned at least one survey, with 596 returning a baseline survey and 494 returning a follow-up survey. The majority of teachers were female (84%), had a permanent full-time teaching position (85%) and taught only primary grades (grades 3–6; 54%). The mean age of teachers was 40 years, and on average teachers had 14 years of teaching experience. Ninety four in-school champions returned a survey. Teacher characteristics are detailed in Table 4.

**Table 4:** Results from linear mixed models illustrating differences in minutes of physical activity scheduled by teachers from baseline compared to 12-month follow-up

Outcome	Baseline Mean (SD)	12-month follow-up Mean (SD)	Unadjusted Mean difference (95% CI)	Adjusted Mean* difference (95% CI)	*p*-value
Total physical activity	124.0 (46.4)	152.8 (45.8)	25.5 (20.4, 30.6)^**a**^	26.8 (21.2, 32.4)^**b**^	< 0.001
Energisers	17.0 (26.4)	38.1 (29.9)	19.1 (15.4, 22.8)^**a**^	20.6 (16.5, 24.6)^**b**^	< 0.001
Integrated lessons	11.2 (19.0)	15.7 (18.8)	4.7 (1.6, 7.8)^**c**^	4.6 (1.4, 7.8)^**c**^	0.006
PE	47.8 (32.3)	51.5 (34.2)	3.8 (-0.2, 7.8)^**a**^	2.7 (-1.8, 7.1)^**b**^	0.24
Sport	53.3 (24.8)	54.0 (26.2)	1.2 (-2.0, 4.3)^**a**^	1.3 (-2.0, 4.6)^**b**^	0.43

*Adjusted for: school factors: type (i.e. government, Catholic, independent), geographical location and socioeconomic disadvantage classification; teacher factors: sex, employment status, years of teaching experience and whether they job share

^a^Number of schools = 88, which includes 84 schools with valid baseline data and 73 schools with valid follow-up; total number of teachers = 663

^b^Number of schools = 88, which includes 84 schools with valid baseline data and 73 schools with valid follow-up; total number of teachers = 591

^c^Unadjusted analysis: number of schools = 85, which includes 75 schools with valid baseline data and 65 with valid follow-up, total number of teachers = 405; Adjusted analysis: number of schools = 85, total number of teachers = 397. NB: There fewer schools included in the analysis of integrated lessons due to missing/invalid data for this component of physical activity

#### Dose delivered and adherence of the implementation strategies (i.e. PACE)

Of the 100 schools that were involved in the PACE evaluation, > 50% adhered to all components of the following implementation strategies: centralize technical assistance and provide ongoing consultation, mandate change, identify and prepare champions, and develop a formal implementation blueprint (see Table 1). Of the other three implementation strategies adherence was > 75% for most of the components except for one each (see Table 1).

#### Sustainability (maintenance)

From surveyed principals at follow-up (*n* = 54), when asked about ongoing executive support, 100% indicated that they would continue to support teachers at their school to schedule 150 min of physical activity. When asked about ongoing support of the strategies that make up PACE, 98% (*n* = 52) of principals indicated that they would continue to utilise the role of the in-school champion, and 76% (*n* = 41) indicated that they would continue to provide the PACE resources to teachers. From surveyed in-school champions, when asked about their ongoing support, 93% (*n* = 75) indicated that they would continue to support the scheduling of physical activity by teachers at their school in the future. Furthermore, 63% (*n* = 52) indicated that they had measures in place to handover their role to a new in-school champion if they were to leave the school.

### Implementation determinants

#### Acceptability of implementation strategies (i.e. PACE)

The percentage of teachers that agreed or strongly agreed that the implementation strategies were acceptable in assisting them to schedule classroom physical activity ranged from 51 to 78% (see Table 5).

**Table 5:** Teachers’ perceived acceptability of the implementation strategies

PACE strategy	Survey item(s) used to assess acceptability	Agree/Strongly agree n (%)
Mandate change	I have support from my school executive to implement PACE	339 (76%)
Identify and prepare in-school champions	I have support from my in-school champion to implement PACE	276 (77%)
	The implementation of the in-school activities by my in-school champion was acceptable in assisting me to schedule physical activity in my class	254 (73%)
	The assistance I receive from my in-school champion was acceptable	261 (75%)
Develop a formal implementation blueprint	The physical activity plan developed by the in-school champion was acceptable in assisting me to schedule physical activity in my class	226 (66%)
Conduct educational outreach visits	The whole school staff meeting was acceptable in assisting me to schedule physical activity in my class	271 (78%)
	The information I received at the whole school meeting was acceptable in assisting me to schedule physical activity in my class	271 (78%)
Develop and distribute educational materials	The support strategies were appropriate in assisting me to schedule physical activity in class	341 (78%)
	The support strategies were easy to use in assisting me to schedule physical activity in my class	341 (78%)
	The information on the online portal was acceptable	177 (51%)
Change physical structure and equipment	The equipment pack was acceptable in assisting me to schedule physical activity in my class	259 (61%)

#### Cost and cost effectiveness of implementation strategies (i.e. PACE)

The total cost of PACE was AUD$168,251, with a mean cost per school of AUD$1,047 (UI $1,020, $1,073). This included costs incurred by the health service with regards to delivering the PACE strategies (total $99,711), as well as costs incurred by schools for engagement with PACE strategies, including staff meetings, emails and work on relevant documents (total $68,540). It was not feasible to collect data regarding the costs associated with usual practice. We assumed that all costs associated with PACE were wholly incremental to usual practice.

**Fig. 1:**
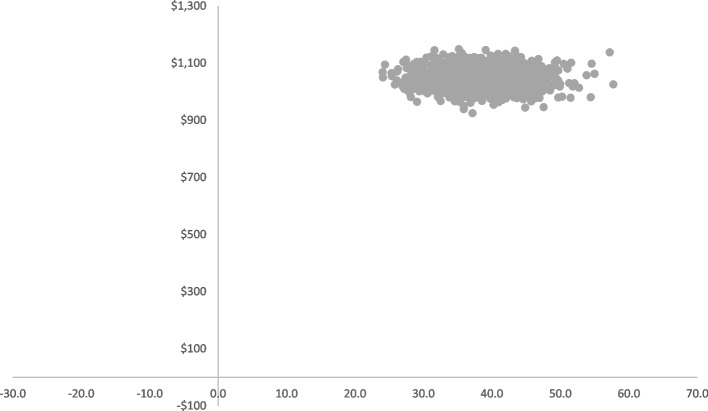
Cost effectiveness plane illustrating the joint distribution between the total minutes of physical activity scheduled by classroom teachers and the cost of PACE for the bootstrapped replications

The incremental cost per additional minute of physical activity scheduled based on the paired cost and outcome data (i.e. total minutes of scheduled physical activity per week), was calculated to be AUD$27 per school (UI $24, $31). The joint distribution of the total minutes of physical activity scheduled by classroom teachers per week and the cost of PACE from the bootstrapped replications are shown in Fig. 1. All values are positioned in the upper right quadrant of the cost effectiveness plane, indicating that PACE is more effective than usual care but delivery is at a higher cost.

## Discussion

This study illustrates one of few attempts to scale-up an effective multi-strategy implementation intervention to a large number of schools across multiple regions. The findings suggest that the PACE strategy was effective in improving policy implementation, and was well delivered. The findings provide useful insights for policy makers and practitioners responsible for supporting student health at a population level through the implementation of school-based policies.

In this study, PACE was successful in achieving an almost 30 min increase per week in teachers’ scheduling of physical activity from baseline compared to 12-month follow-up. The effect was smaller than what was achieved in the original pilot [17] and implementation trial [14], which recorded improvements of approximately 37 and 45 min, respectively. Effect attenuation (i.e. voltage drop), however, is not uncommon as interventions are scaled-up. For example, a 2019 systematic review of scaled-up physical activity interventions in community settings, found that when scaled-up physical activity interventions typically achieve half of their pre-scale effect sizes [23]. This study found that effect attenuation also occurs on measures of implementation outcomes and is consistent with other studies [24]. Smaller improvements in implementation outcomes at scale is of concern as they are likely to yield smaller improvement in student physical activity and associated health outcomes. Further research is required to better understand why this occurs, and to identify strategies to prevent or reduce the magnitude of any effect attenuation.

Data from this study found the implementation strategies achieved broad reach and high adherence and dose ratings for most elements. However, adherence by teacher’s were <50% to the following components: accessing professional learning videos, capturing and sharing local knowledge, and developing classroom physical activity packs. Such findings suggest better adherence of these strategies by schools may improve the impact of PACE on teacher’s scheduling of classroom physical activity. Future adaptations should look into attempting to engage teachers in using online resources by embedding such resources into existing online tools that they already access [32]. Encouragingly, both principals and in-school champions indicated their ongoing commitment to supporting teachers to schedule physical activity in line with the NSW policy requirements.

PACE had an average incremental cost of AUD$1,047 per school to deliver. There is a lack of studies evaluating the cost of implementation interventions [33], particularly those focused on physical activity in school, making it difficult to assess the cost implications of PACE. However, the cost does seem favourable when compared to scale-up of an implementation intervention designed to support the delivery of physical activity in secondary schools located in economically disadvantaged areas, which had a higher average cost of delivery at $17,296 per school [34]. However, due to the differences in setting (primary schools vs. economically disadvantaged secondary schools) it is difficult to make direct comparisons between these two implementation interventions. As cost is an important determinant of implementation and scale-up future efforts should be undertaken to explore whether the cost of PACE could be further reduced while preserving the positive impact of the implementation strategy. For instance alternate modes of delivery of some of the strategies, such as changing the mode of delivery for workshops from in-person to online (i.e., via a self-directed delivery platform), may be worthy of future investigation. In fact, the research team have recently employed such adaptations and investigated the effect of a version of PACE that removes some of the components with a higher cost. This study found the lower cost version was similarly effective on increasing teacher’s scheduling of classroom physical activity [30].

### Limitations

There are several limitations that should be considered when interpreting the results of this study. First the uncontrolled before and after design is considered a less robust research design for assessing the effectiveness of an intervention, due to the possible impact of confounding and bias. However, it is considered an acceptable pragmatic design to assess the effect of scaling up an intervention with pre-existing evidence of effectiveness [35]. Second, the self-report nature of the primary outcome is subject to social desirability and other reporting biases. However, it is the same measure used in all previous randomised trials and it is not feasible to obtain an objective measure of teachers scheduling of physical activity across such a large number of schools. Third, while we met our target of delivering PACE to 100 schools this represented only 26% of eligible schools. While we stopped active recruitment once we hit our target sample size additional work to improve the reach and further scaling of PACE to more schools across NSW is needed to ensure the benefits are experienced at a population level. Fourth, we did not set out to assess or compare the differences in outcomes across regions. The main aim was to evaluate whether PACE could be successfully scaled across multiple regions at one time while maintaining a positive impact. It is possible that differences in regional characteristics or infrastructure could impact on how PACE is delivered and received across different regions. However, sensitivity analysis controlling for region did not significantly change the overall findings or conclusions, although we recognise this analysis does not inform us of any potential differential effects by region. Future research should explore whether the delivery and impact of PACE differs based on region, as this may inform whether tailoring or adaptations are required. Finally, while these findings are encouraging there are aspects of PACE that could be improved that could assist with future scale-up efforts. In particular several of the individual implementation strategies were poorly adhered to by schools. In particular strategies that required additional resources (e.g., creating equipment packs for all classes) or unallocated time commitments (e.g., attending a follow-up meeting, and accessing online learning videos). Strategies that require personal commitment or resource, regardless how small, may not be suitable for this population.

## Conclusions

PACE is an effective and acceptable implementation intervention for supporting teachers to increase their scheduling of classroom physical activity. Future efforts should be made to reduce the cost and improve the reach of PACE. Investigating different modes of delivery for some of the most resource intensive strategies is one suggested avenue for improving this implementation intervention. Through fine tuning and further improving the scalability of PACE we have an opportunity to improve schools’ physical activity policy requirements, and more importantly the health and wellbeing of school children.

### Supplementary Information

**Additional file 1: Supplementary Table 1.** Results from sensitivity analysis of linear mixed models illustrating differences in minutes of physical activity scheduled by teachers from baseline compared to 12-month follow-up for only those schools contributing valid data for both baseline and follow-up. *Adjusted for: school factors: type (i.e. government, Catholic, independent), geographical location and socioeconomic disadvantage classification; teacher factors: sex, employment status, years of teaching experience and whether they job share. ^a^Does not include random slope for time to ensure model fit. ^b^Number of schools = 69; number of teachers with valid data for unadjusted model = 601; number of teachers with valid data for adjusted model = 534. ^c^Number of schools = 68; number of teachers with valid data for unadjusted model = 372; number of teachers with valid data for adjusted model = 365. **Supplementary Table 2.** Results from sensitivity analysis of linear mixed models illustrating differences in minutes of physical activity scheduled by teachers from baseline compared to 12-month follow-up also controlling for region. *Adjusted for: school factors: type (i.e. government, Catholic, independent), geographical location and socioeconomic disadvantage classification; teacher factors: sex, employment status, years of teaching experience and whether they job share and region. ^a^Number of schools = 88; number of teachers = 591. ^b^Number of schools = 85; number of teachers = 397

## Data Availability

The datasets used for the current study are available from the corresponding author upon reasonable request.

## Abbreviations

MVPA: Moderate-to-vigorous physical activity
AUD: Australian dollar
WHO: World Health Organization
NSW: New South Wales
PE: Physical Education
PACE: Physically Active Children in Education
RCT: Randomised controlled trial
CI: Confidence interval
ICC: Intraclass correlation coefficient
UI: Uncertainty interval
