# Supplementary material for: Evaluating the scaling up of an effective implementation intervention (PACE) to increase the delivery of a mandatory physical activity policy in primary schools
Source: Int J Behav Nutr Phys Act. 2023 Sep 6;20:106. doi: 10.1186/s12966-023-01498-y (PMC10481546; doi:10.1186/s12966-023-01498-y)
Supplement: Supplementary file 1 — Additional file 1: Supplementary Table 1. Results from sensitivity analysis of linear mixed models illustrating differences in minutes of physical activity scheduled by teachers from baseline compared to 12-month follow-up for only those schools contributing valid data for both baseline and follow-up. *Adjusted for: school factors: type (i.e. government, Catholic, independent), geographical location and socioeconomic disadvantage classification; teacher factors: sex, employment status, years of teaching experience and whether they job share. aDoes not include random slope for time to ensure model fit. bNumber of schools = 69; number of teachers with valid data for unadjusted model = 601; number of teachers with valid data for adjusted model = 534. cNumber of schools = 68; number of teachers with valid data for unadjusted model = 372; number of teachers with valid data for adjusted model = 365. Supplementary Table 2. Results from sensitivity analysis of linear mixed models illustrating differences in minutes of physical activity scheduled by teachers from baseline compared to 12-month follow-up also controlling for region. *Adjusted for: school factors: type (i.e. government, Catholic, independent), geographical location and socioeconomic disadvantage classification; teacher factors: sex, employment status, years of teaching experience and whether they job share and region. aNumber of schools = 88; number of teachers = 591. bNumber of schools = 85; number of teachers = 397 [file 12966_2023_1498_MOESM1_ESM.docx]

**Supplementary Table 1. Results from sensitivity analysis of linear mixed models illustrating differences in minutes of physical activity scheduled by teachers from baseline compared to 12-month follow-up for only those schools contributing valid data for both baseline and follow-up**

| **Outcome** | **Baseline**  **Mean (SD)** | **12-month follow-up**  **Mean (SD)** | **Unadjusted**  **Mean difference (95% CI)** | **Adjusted Mean***  **difference (95% CI)** | **p-value** |
| --- | --- | --- | --- | --- | --- |
| Total physical activity^b^ | 125.2 (SD=46.4) | 152.6 (SD=45.3) | 25.3 (20.1, 30.5) | 26.0 (20.3, 31.7) | <0.001 |
| Energisers^b^ | 18.0 (SD=27.0) | 37.6 (SD=29.6) | 18.1 (14.3, 21.9) | 19.8 (15.7, 24.0)**^a^** | <0.001 |
| Integrated lessons^a^ | 11.6 (SD=19.4) | 15.6 (SD=18.6) | 4.3 (1.1, 7.5) | 3.9 (0.5, 7.2) | 0.024 |
| PE^b^ | 47.7 (SD=31.6) | 51.8 (SD=34.6) | 4.1 (0.1, 8.2) | 2.7 (-1.9, 7.2) | 0.24 |
| Sport^b^ | 53.2 (SD=24.6) | 54.2 (SD=26.2) | 1.5 (-1.7, 4.6) | 1.6 (-1.7, 5.0) | 0.34 |

*Adjusted for: school factors: type (i.e. government, Catholic, independent), geographical location and socioeconomic disadvantage classification; teacher factors: sex, employment status, years of teaching experience and whether they job share

^a^Does not include random slope for time to ensure model fit

^b^Number of schools = 69; number of teachers with valid data for unadjusted model = 601; number of teachers with valid data for adjusted model = 534

^c^Number of schools = 68; number of teachers with valid data for unadjusted model = 372; number of teachers with valid data for adjusted model = 365

**Supplementary Table 2. Results from sensitivity analysis of linear mixed models illustrating differences in minutes of physical activity scheduled by teachers from baseline compared to 12-month follow-up also controlling for region**

| **Outcome** | **Baseline**  **Mean (SD)** | **12-month follow-up**  **Mean (SD)** | **Adjusted Mean***  **difference (95% CI)** | **p-value** |
| --- | --- | --- | --- | --- |
| Total physical activity^b^ | 124.0 (46.4) | 152.8 (45.8) | 27.0 (21.4, 32.6) | <0.001 |
| Energisers^b^ | 17.0 (26.4) | 38.1 (29.9) | 20.7 (16.6, 24.8) | <0.001 |
| Integrated lessons^a^ | 11.2 (19.0) | 15.7 (18.8) | 4.6 (1.4, 7.9) | 0.006 |
| PE^b^ | 47.8 (32.3) | 51.5 (34.2) | 2.7 (-1.7, 7.2) | 0.23 |
| Sport^b^ | 53.3 (24.8) | 54.0 (26.2) | 1.4 (-1.9, 4.6) | 0.41 |

*Adjusted for: school factors: type (i.e. government, Catholic, independent), geographical location and socioeconomic disadvantage classification; teacher factors: sex, employment status, years of teaching experience and whether they job share and region

^a^Number of schools = 88; number of teachers = 591

^b^Number of schools = 85; number of teachers = 397
